# An Energy-efficient Rate Adaptive Media Access Protocol (RA-MAC) for Long-lived Sensor Networks

**DOI:** 10.3390/s100605548

**Published:** 2010-06-02

**Authors:** Wen Hu, Quanjun Chen, Peter Corke, Damien O’Rourke

**Affiliations:** 1 ICT Centre, CSIRO, Brisbane 4069, Australia; E-Mails: peter.corke@csiro.au (P.C.); damien.o’rourke@csiro.au (D.O.R.); 2 School of Computer Science and Engineering, The University of New South Wales, Sydney 2052, Australia; E-Mail: quanc@cse.unsw.edu.au

**Keywords:** wireless sensor networks, energy efficient, rate adaptive, performance evaluation

## Abstract

We introduce an energy-efficient Rate Adaptive Media Access Control (RA-MAC) algorithm for long-lived Wireless Sensor Networks (WSNs). Previous research shows that the dynamic and lossy nature of wireless communications is one of the major challenges to reliable data delivery in WSNs. RA-MAC achieves high link reliability in such situations by dynamically trading off data rate for channel gain. The extra gain that can be achieved reduces the packet loss rate which contributes to reduced energy expenditure through a reduced numbers of retransmissions. We achieve this at the expense of raw bit rate which generally far exceeds the application’s link requirement. To minimize communication energy consumption, RA-MAC selects the optimal data rate based on the estimated link quality at each data rate and an analytical model of the energy consumption. Our model shows how the selected data rate depends on different channel conditions in order to minimize energy consumption. We have implemented RA-MAC in TinyOS for an off-the-shelf sensor platform (the TinyNode) on top of a state-of-the-art WSN Media Access Control Protocol, SCP-MAC, and evaluated its performance by comparing our implementation with the original SCP-MAC using both simulation and experiment.

## 1. Introduction

In this paper, we present an energy-efficient Rate-Adaptive MAC (RA-MAC) algorithm for long-lived WSNs [1–3]. The lifetime of a long-lived WSN is typically expected to be on the order of years with node duty cycles less than 1% (e.g., less than 1 sample per minute). (An earlier version of this article appeared as a poster abstract in Proceedings of the 6th European Conference on Wireless Sensor Networks (EWSN 09) [4]. This article features comprehensive design, analysis, implementation, and evaluation of proposed Rate Adaptive Media Access Protocol.)

Performance of radio communication links, in terms of Packet Reception Rate (PRR) for example, are critical to the overall performance of wireless sensor networks, both in terms of network data throughput and energy consumption. Higher layers of the network stack are designed to maximally utilize the communications link and thus require an understanding of link characteristics.

A long-term water monitoring network [3] of 8 nodes monitors ground water extraction and salinity in a sugar cane growing region of north-eastern Australia. The network is unusual in that it has long radio links—in the range from 1 to 1.8 km. In late summer, researchers observed that many of the radio links go down at night, regularly. They observed no direct correlation with local climatic conditions such as precipitation or humidity measured at a nearby weather station, and speculate the problem is due to a layer of moist air above the fully grown cane which is trapping or reflecting the radio waves. In an independent study [5], detailed work shows that the transmission range of Mica2 motes can be reduced by a factor of 5 in high humidity conditions, including fog.

Zamalloa and Krishnamachari [6] take a theoretical approach to the problem. They model the radio channel, the radio itself, and the noise floor, and show the existence of a transition region, also referred to as a “gray area” [7], characterised by highly variable packet reception rates. They show how the transition region limits can be determined, and that these limits are a function of the received Signal-to-Noise Ratio (SNR).

Traditional approaches to compensate for poor link quality in WSNs use sophisticated MAC and routing protocols with acknowledgements and retransmissions. However, these techniques introduce extra traffic, increased energy consumption, and increase the size and complexity of the node’s software.

There has been much less work focused on addressing the underlying link quality. Well known approaches in the communications field include: relay nodes, higher transmit power, delay tolerant routing, frequency diversity, antenna diversity, and spread spectrum modulation techniques. All have difficulties or cost implications.

Relay nodes allow for shorter links with higher quality, but are expensive. For example, the transmission range of the Mica2 motes can be reduced by a factor of 5 in high humidity conditions [5]. To compensate for this using relay nodes would require 5 × 5 = 25 times as many nodes to be deployed over the same area. Along with the extra cost of the hardware involved, it has been noted (e.g., [2]) that multi-hop transmissions may be more energy expensive compared with single-hop transmissions.

Transmission power is limited by regulation and most nodes already operate at the maximum permissible. Delay tolerant architectures can buffer data until the link quality improves but at the expense of increased latency and energy issues (perhaps extra flash read/write operations [2]). Increasing the channel gain can be achieved by larger, or more directional antennas. However, large antennas go against the ultimate goals of wireless sensor networks, in general, and their increased cost can introduce scalability issues. Moreover, directional antennas do not suit many network topologies.

Coding and data bit rates are the *only remaining degrees* of freedom in this problem. In general, users accept the data rates offered to them by the radio chip rather than specifying the data rate required for the particular link. While links close to a root node may have a requirement for considerable throughput, those closer to the edge of the network rarely need the rate provided by the radio, e.g., 50 kbps for the Fleck-3B or 250 kbps for IEEE 802.15.4 compliant radios. With suitable coding we could, for example, halve the data bit rate and achieve 3 dBm of extra gain (see Section 3.). The resulting increase in SNR could move us from the transition zone to the region of good reception, significantly reducing the requirement for packet retransmissions. We can therefore achieve better link performance with less energy consumption by trading off what we do not actually need: high data bit rates. This is the fundamental premise of our new rate adaptive data-link algorithm, RA-MAC: we dynamically trade data bit rate to improve the link quality, rather than use retransmissions. The RA-MAC approach therefore *reduces the number of transmissions*, reduces channel utilization and *lowers the energy* required to send messages within the network.

The contributions of this paper include:

- RA-MAC, which is, to the best of our knowledge, the ***first rate-adaptive Media Access Protocol*** for WSNs with the purpose of ***minimizing network energy consumption***. In contrast to conventional Wireless Local Area Networks (WLANs), where optimizing link throughput is the main focus, node energy consumption is typically a more important performance metric in WSNs. Further, rate-adaptive protocols in WLANs are typically deeply coupled with 802.11 Request To Send (RTS) and Clear to Send (CTS) message exchanges, but the packets are typically too small in WSNs to justify the RTS/CTS exchanges (e.g., the default MAC payload size is 28 bytes in TinyOS 2.x). Moreover, WLAN MAC typically do not take *the cost of acknowledgement packets* into account because the sizes of the data packets are significantly larger than the acknowledgement packets.
- An analytical model of the energy consumption under different channel conditions, which we use to optimize the overall network energy consumption.
- Evaluation of RA-MAC using both simulation and experiment demonstrates the performance of the protocol. We have demonstrated the feasibility of our RA-MAC protocol using TinyOS [8] running on the off-the-shelf TinyNode platform [9].

The rest of the paper is organized as follows. We present related work in Section 2. We discuss our hypothesis regarding adaptive data rates in Section 3. and set up an analytical model to optimize node energy consumption under different channel conditions in Section 4. We present the details of the proposed RA-MAC protocol in Section 5. Section 6. presents the experimental results that compare the performance of RA-MAC to that of SCP-MAC [10], followed by the simulation comparison between a conventional WLAN rate adaptive algorithm and the proposed RA-MAC in Section 7. Finally, we present our conclusions in Section 8.

## 2. Related Work

In this section, we discuss the related MAC research for both wireless sensor networks and conventional wireless local area networks.

### 2.1. Medium Access Control Protocol in WSNs

Energy-efficiency is one of the most important performance metrics in WSNs because sensor nodes typically have a very limited energy supply and are expected to operate independently over a long time-period. This is a significant differentiator between research in WSN MAC and WLAN MAC. Because the traffic load in WSNs is typically low, an effective method to reduce the (idle listening) duty cycles of nodes is of prime importance.

S-MAC is a classic CSMA-style protocol for sensor networks [11]. Nodes are coarsely time synchronized and transmit using a fixed wake-up schedule in a multi-hop network to save energy. S-MAC enables the nodes to run at duty cycles of 1 – 10%.

T-MAC improves the performance of S-MAC by using an adaptive timer that can adjust the wake-up schedule of a node dynamically based on traffic fluctuations [12].

A Low Power Listening (LPL) node periodically and briefly samples the channel for the presence of radio transmissions [13]. The node powers down immediately if no transmission is detected. Otherwise, the node keeps listening until a short transmission start symbol is detected. B-MAC [14] is the LPL implementation in TinyOS [8] and has a well-defined interface that allows applications to control sleep intervals, back-off window size, and power down policy within the MAC. It uses an advanced clear channel assessment (CCA) algorithm to handle random channel noise.

As an alternative to transmitting a long preamble, which creates overhearing overhead for the neighboring nodes, X-MAC [15] sends out a strobe of small packets, which includes the destination node address. This enables faster power-down reducing the listening duty-cycles for unintended recipients. F-MAC [16] sends each packet as a number of frame-lets. A carefully chosen frame-let frequency of each node can guarantee collision-free transmission of at least one frame-let.

CSMA-style protocols provide good system performance, such as throughput, when the traffic load is low. However, when the network approaches a congested state, the performance of CSMA-style protocols degrades dramatically. Therefore, a class of MAC protocols based on TDMA [2,10,17–19] have been proposed. The conventional TDMA protocols require a centralized entity to assign transmission slots, which is not feasible for large scale sensor networks. Luster [2] uses LiteTDMA, a low-power cluster-based MAC protocol, that limits network topology to a single hop.

While TDMA-style MAC protocols can provide better network throughput when the traffic load is high, the complexity of the protocol and the requirement of fine-grained time-synchronization are major disadvantages. Z-MAC [18] is a hybrid CSMA and TDMA scheme. Transmission time slots are assigned to individual nodes in Z-MAC. However, rather than completely owning a time slot as in conventional TDMA, the owner node of the time slot gets transmission precedence by using a smaller competition window than the other nodes.

SCP-MAC [10] addresses the long preamble problem of LPL by introducing scheduled channel polling to LPL. Nodes are fine-grained time synchronized in SCP-MAC. Instead of sending out a long preamble, a node sends a very short “tone” to wake up receivers. Therefore, SCP-MAC can lower node duty cycle to less than 1%— a factor of 10 reduction compared with both S-MAC and B-MAC.

While a number of adaptive mac algorithm exist for wireless sensor networks they are inherently different by design in that most adapt to varying traffic patterns. For example Demand Wakeup MAC (DW-MAC) [20], uses a low-overhead scheduling algorithm that allows nodes to wake up on demand during their sleep period. This adaptively increases the effective channel capacity during an operational cycle as traffic load increases. Convergent MAC (CMAC) [21] avoids synchronization overhead but still supports low latency. By avoiding communication when there is no traffic, CMAC allows for very low-duty cycles. However, when carrying traffic, CMAC begins using unsynchronized duty cycling anycast transmissions to wake up forwarding nodes, and then converges to unicast, synchronized scheduling.

### 2.2. Rate Adaptive MAC in Wireless Local Area Networks (802.11)

A number of rate adaptation algorithms [22–25] have been proposed in the area of Wireless Local Area Networks (WLANs) in the last few years. The basic idea is to increase the data rate upon consecutive transmission successes and to use probe packets to access new transmission rates. While all these algorithms seem logically correct, little is known about their practical performance.

Auto Rate Fallback (ARF) [22] was the first published rate adaption algorithm in WLAN, and has been implemented commercially. ARF increases the transmission data rate when a sender receives acknowledgements for a number of consecutive packets, and decreases transmission data rate when a sender fails to receive acknowledgements. Instead of using a fixed number of consecutive successful transmissions to determine the increase (or decrease) in data rates, Adaptive Auto Rate Fallback (AARF) [23] uses an adaptive number of consecutively successful transmissions by a binary exponential back-off algorithm.

Receiver Based Auto Rate (RBAR) [24] considers the problem of asymmetric wireless links and uses the channel quality information, e.g., SNR, at the receiver to select a data rate. Instead of using a fixed maximum message size, Opportunistic Auto Rate (OAR) [25] attempts to exploit the wireless channel whenever the link quality is good by allowing higher data rate transmissions to use larger message sizes. Therefore, OAR can provide better throughput. Haratcherev et al. [26] propose a hybrid rate adaptive approach based on both SNR and delivery rate statistics for streaming applications in ***single-hop*** WLANs. Our work is closest to MiSer [27] because both algorithms try to minimize energy consumption by adapting the transmission rates at the physical layer. However, there are two key differences between RA-MAC and MiSer. First, MiSer is heavily reliant on the IEEE 802.11b MAC protocol (*i.e.*, Request To Send (RTS), Clear To Send (CTS), data, and acknowledgement). However, the packets in WSNs are typically considered *too small to justify RTS/CTS exchanges*. Second, MiSer does not take *the cost of acknowledgement packets* into account because the sizes of the data packets are significantly larger than the acknowledgement packets. However, because the data packets in sensor networks are typically small, previous research [28] shows that it is important to consider the cost of acknowledgement packets. As show in [27], MiSer is most suitable for data transmissions with large data payloads (e.g., 1,500 bytes). On the other hand, RA-MAC is optimized to work with tiny payloads (e.g., 34 bytes) in WSNs.

### 2.3. Summary

Previous Media Access Control (MAC) protocol research in sensor networks has not considered the design space where data rate and link reliability can be traded off. We argue that the proposed adaptive data rate MAC algorithm can be added to existing protocols to further optimize their performance. Previous adaptive MACs for WLANs are designed to ***optimize network throughput*** rather than energy efficiency, are deeply coupled with the IEEE 802.11 MAC (e.g., ***RTS/CTS exchanges***), and do not take acknowledgement packets into accounts. RA-MAC, to the best of our knowledge, is ***the first MAC protocol*** to consider adaptive data rates in sensor networks, which have unique challenges such as network energy consumption, lack of RTS/CTS exchanges, and relatively significant acknowledgement packet sizes.

## 3. Packet Reception Rate

Let us first introduce the necessary mathematical notation, all of which is collectively listed in Table 1. Wireless link quality, e.g., PRR, is a function of Bit Error Rate (BER) and the packet size in digital communications; namely,

(1) $$\mathrm{PRR}={\left(1-\mathrm{BER}\right)}^{f_{\mathrm{data}}}$$

where *f_data_* is the data frame size in bits, and

(2) $$\mathrm{BER}=f\;\left(\frac{E_{b}}{N_{0}}\right)$$

where *E_b_/N*_0_ is the ratio of the average energy per bit to the single-sided noise power spectral density. Signal to Noise Ratio (SNR) can be calculated as

(3) $$\mathrm{SNR}=\frac{E_{b}}{N_{0}}\frac{R}{B}$$

where *R* is the data bit rate and *B* is the channel bandwidth. Note that, by definition, SNR is the ratio of received signal strength to the channel noise power, *i.e.*, *SNR* = *RSSI/N*. Therefore, Received Signal Strength Indicator (RSSI) is

(4) $$\mathrm{RSSI}=\mathrm{SNR}\cdot N=\frac{E_{b}}{N_{0}}\frac{R}{B}N$$

where *N* is channel noise power, which can be calculated as

(5)  N=kTB,

where *k* is Boltzmann’s constant and *T* is the effective temperature in degrees Kelvin. For simplicity we ignore receiver system noise generated in the amplification and processing of the signal, which is usually constant.

By substituting Equation (5) into Equation (4), and re-arranging the terms, we have

(6) $$\frac{E_{b}}{N_{0}}=\frac{\mathrm{RSSI}}{\mathrm{kTR}}$$

Since *k*, *T* are constants, given a specific modulation format (e.g., two-level FSK), *E_b_/N*_0_ only depends upon the RSSI and data rate *R*. Recall that Bit Error Rate (BER) is a function of *E_b_/N*_0_ (see Equation (2)) and PRR is again a function of BER (see Equation (1)). Consequently, PRR is a function of RSSI and *R*. We have

(7) $$\mathrm{PRR}=g(\frac{\mathrm{RSSI}}{R})$$

## 4. Energy Consumption Analysis

We use a network energy consumption model similar to [10], in which a node’s power consumption is different for the listening (*P_l_*), transmitting (*P_tx_*) and receiving (*P_rx_*) states.

(8) $$E(R)=P_{l}t_{l}+P_{\mathrm{tx}}(t_{\mathrm{tone}}+\frac{f}{R})+nP_{\mathrm{rx}}(t_{\mathrm{tone}}+\frac{f}{R})$$

where *t_l_*, *t_tone_* are channel listening and tone sending duration respectively, *f* is packet frame size in bits, and *n* the number of neighbors. MAC protocols such as [15] feature neighbor overhearing avoidance and *n* becomes one, which is a special case of Equation (8). We ignore node sleep power consumption because it is typically less than 0.1% of transmitting or receiving power consumption.

***Link layer hop-by-hop packet retransmission*** is typically used in WSNs to increase end-to-end delivery reliability [28,29]. Taking the size of the acknowledgement packet (*f_ack_*) and possible asymmetric link quality into account, we can rewrite Equation (8) in terms of the data transmission energy and the ACK transmission energy as

(9) $$E_{\mathrm{data}}(R)=P_{l}t_{l}+P_{\mathrm{tx}}(t_{\mathrm{tone}}+\frac{f_{\mathrm{data}}}{R})+nP_{\mathrm{rx}}(t_{\mathrm{tone}}+\frac{f_{\mathrm{data}}}{R})$$

and

(10) $$E_{\mathrm{ack}}(R)=P_{\mathrm{tx}}\frac{f_{\mathrm{ack}}}{R}+nP_{\mathrm{rx}}\frac{f_{\mathrm{ack}}}{R}$$

For a given data rate *R*, it takes *α /* (*PRR*_data_*PRR*_ack_) data packet transmissions and *α / PRR*_ack_ ACK packet transmissions to achieve the required link reliability *α. PRR*_data_ is the packet reception rate of a data frame, and *PRR*_ack_ is the packet reception rate of an ACK frame. Then, the energy consumption is

(11) $$E(R,\;\mathrm{RSSI})=\frac{\alpha }{{\mathrm{PRR}}_{\text{data}}{\mathrm{PRR}}_{\text{ack}}}E_{\mathrm{data}}(R)+\frac{\alpha }{{\mathrm{PRR}}_{\text{ack}}}E_{\mathrm{ack}}(R)$$

Note that *PRR* is a function of *RSSI* and data bit rate *R* (Equation (7)). To select the optimal data rate that minimizes energy consumption, we can express the problem as

(12) $$\hat{R}=\underset{R}{\text{argmin}}\;\;E(R,\;\mathrm{RSSI})$$

**Figure 1.**
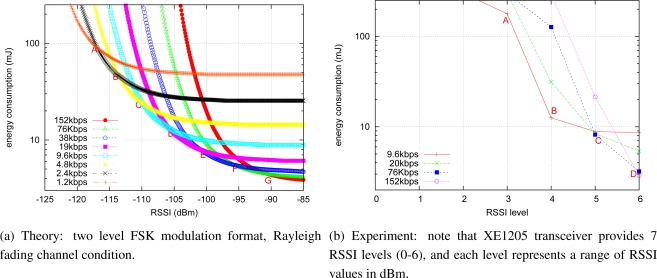
Network energy consumption *vs.* RSSI values at different data rates. (Note the Y-axis log scale).

For a limited number of data rates *R*, it is easy to calculate the network’s energy consumption by Equation (11), and to select the minimum value. Given *α* = 1 and *n* = 10, the network energy consumption at different data rates is shown in Figure 1(a) plotted against RSSI. The parameters are for the Semtech XE1205 transceiver used in our experiments and are listed in Table 1. Points *A*, *B*, ..., *G* are the network energy consumption equilibrium points for two neighboring data rates. They can be used to select an optimal data rate, to minimize network energy consumption, for a given RSSI value. For example, when *A < RSSI < B*, data transmission rate *R* = 2.4 kbps will achieve minimum network energy consumption.

To verify the analysis of Equation (11) and (12), we performed an experiment with off-the-shelf TinyNodes over an indoor link. In the experiment, the transmitter sent packets to the receiver at 5 Hz with 4 different data rates (9.6, 20, 76, and 152 kbps). Each experiment lasted for 15 minutes, and we repeated the same experiment 5 times. Then, we computed the energy consumption for the experiments of using the parameters listed in Table 1. The results shown in Figure 1(b) shows a similar trend to Figure 1(a). The ***Semtech XE1205 transceiver provides only 7 RSSI levels (0–6)*** so the number of decision points in Figure 1(b) is smaller than for Figure 1(a). For example, if *RSSI* = 5 (Point C in the Figure), *R* = 76 kbps consumes the minimum network energy according to Figure 1(b).

Although we can in theory choose the optimal data rate to minimize network energy consumption according to Equation (11) and (12), in practice they are difficult to obtain because of their dependence on channel condition which is unknown in advance and dynamic over time. Therefore, in the next section, we will introduce an adaptive MAC protocol, which is tailored to handle channel dynamics for the purpose of selecting an optimal data rate.

## 5. Rate Adaptive MAC (RA-MAC)

In this section, we present details of our proposed Rate Adaptive Media Access Control (RA-MAC) scheme for sensor networks.

Based on Equation (11) and (12), we can select the optimal data rate to minimize network energy consumption given full knowledge of the channel condition. However, it is very difficult, if not impossible, to collect this information in advance and as discussed earlier it is quite dynamic, particularly if operating within the transition region. Therefore, we need to continually monitor the channel condition (e.g., *n*, *RSSI*, *PRR*_data_, and *PRR*_ack_) and change the transmission data rate adaptively. The number of neighbors (*n*) can be easily obtained by overhearing neighbor’s communications traffic. The value of the remaining parameters (*RSSI*, *PRR*_data_, and *PRR*_ack_) are time varying and RA-MAC includes algorithms to estimate them on-line in order to select the optimal transmission data rates.

Algorithm 1 is used to send a data packet. The sender node estimates the received signal strength ( $\hat{\mathrm{RSSI}}$) of the current packet (note that RSSI is measured at the receiver). This is based on a weighted moving average filter over previous RSSI values that were piggybacked on previous ACK packets (More sophisticated estimates could include auto-regressive terms, neural networks or even a Kalman filter. However, such algorithms are typically computationally intensive and challenging to implement on a resource-constrained sensor node. The performance and cost comparison of different estimators is beyond the scope of this paper.). Then, at Line 5, the node calculates the energy consumption based on $\hat{\mathrm{RSSI}}$, *PRR*_data_ and *PRR*_ack_ using Equation (11) for different data rate. The node then chooses the optimal data rate *R* that consumes the least amount of energy (Line 6). Furthermore, in order to adapt faster to the channel condition, once it deteriorates, the node decreases data transmission rate *R* aggressively (Line 12). Similarly, after the channel condition improves, the node increases data transmission rate *R* (Line 8) in order to explore PRR at the higher rate. Here *M* is a protocol parameter. RA-MAC adapts faster to changing channel conditions with smaller *M*. However, a smaller *M* might make RA-MAC overreact to channel noise. We use *M* = 10 in our TinyOS/TinyNode implementation.

**Algorithm 1:** Pseudo-code for transmitting a data packet

1:	**procedure***Transmit*(*Packet*)
2:	**if** Last transmission acknowledged **then**
3:	*S* ⇐ *S* + 1
4:	Estimate $\hat{\mathrm{RSSI}}$: $\hat{\mathrm{RSSI}}⇐(1-\beta_{1})\cdot \hat{\mathrm{RSSI}}+\beta_{1}\cdot {\mathrm{RSSI}}_{\mathrm{prev}}$
5:	Calculate *E*( $\hat{\mathrm{RSSI}}$*, R*) using Equation (11) for different *R*
6:	$R⇐\underset{R}{\text{argmin}}\;\;E(R,\;\mathrm{RSSI})$
7:	**if***S > M***then**
8:	*R* ⇐ *R* + 1
9:	*S* ⇐ 0
10:	**end if**
11:	**else**
12:	*R* ⇐ *R* − 1
13:	*S* ⇐ 0
14:	**end if**
15:	Transmit packet at data rate *R*
16:	**end procedure**

Algorithm 2 shows the pseudo-code for a node after receiving a data packet. The receiver collects RSSI value and data rate *R* from the packet and calculates the Cyclic Redundancy Check (CRC). Then it updates *PRR*_data_(*RSSI*, *R*) using a weighted moving average (Line 5). If the CRC value is correct, the receiver piggybacks *PRR*_data_ value and the RSSI value in the ACK packet and transmits it back to the sender.

Algorithm 3 shows the pseudo-code for a sender waiting to receive an ACK packet. First, the sender sets up a retransmission timer. If the sender receives an ACK before the timer expires, it extracts the real RSSI, and the updated *PRR*_data_ values from the ACK packet (Line 6). Since the ACK packet normally has very small size and does not have a CRC field, we implicitly assume that an ACK packet is always correct if it is received by the sender. Therefore, it is difficult to measure *PRR*_ack_ directly. However, we can use an alternative approach to estimate *PRR*_ack_. We introduce a new parameter named *PRR*_*data*&*ack*_, which is the successful rate of data packet transmission. A data packet transmission is only considered successful when the ACK packet for the data packet is received at the sender. In other words, *PRR*_*data*&*ack*_ is the successful rate of both a data packet and an ACK packet. We have *PRR*_*data*&*ack*_ = *PRR*_data_ · *PRR*_ack_. Therefore, we can estimate *PRR*_ack_ by *PRR*_*data*&*ack*_/*PRR*_data_. In Algorithm 3, Line 14 statistically estimates *PRR*_*data*&*ack*_ using a weighted moving average and then the *PRR*_ack_ is calculated in Line 15.

**Algorithm 2:** Pseudo-code for receiving a data packet

1:	**procedure***ReceiveData*(*Packet*)
2:	Collect *RSSI* and *R*
3:	Calculate CRC
4:	Stat ⇐ (CRC correct) ? 1:0
5:	*PRR*_data_(*RSSI*, *R*) ⇐ (1 − *β*_2_) · *PRR*_data_(*RSSI*, *R*) + *β*_2_ · *Stat*
6:	**if** CRC correct **then**
7:	Transmit ACK packet with RSSI and *PRR*_data_(*RSSI*, *R*)
8:	**end if**
9:	**end procedure**

**Algorithm 3:** Pseudo-code for receiving an ACK packet

1:	**procedure***WaitForAck*( $\hat{\mathrm{RSSI}}$, R)
2:	$\mathrm{RSSI}⇐\hat{\mathrm{RSSI}}$
3:	*t*_0_ ⇐ *t_now_*
4:	**while***t_now_< t*_0_ + *T***do**
5:	**if** ACK received **then**
6:	Stat = 1
7:	*RSSI, PRR*_data_ ⇐ from ACK packet
8:	**end if**
9:	**end while**
10:	**if** ACK not received **then**
11:	Stat = 0
12:	**end if**
13:	*PRR*_*data*&*ack*_ (*RSSI*, *R*) ⇐ (1 − *β*_3_) · *PRR*_*data*&*ack*_ (*RSSI*, *R*) + *β*_3_ · *succ*
14:	*PRR*_ack_(*RSSI*, *R*) ⇐ *PRR_data&ack_*(*RSSI*, *R*)/*PRR*_data_(*RSSI*, *R*)
15:	*RSSI_prev_* ⇐ *RSSI*
16:	**end procedure**

Now we discuss the memory space required in RA-MAC algorithm. The main variables *PRR*_data_, *PRR*_ack_, and *PRR*_*data*&*ack*_ are a function of *RSSI* and *R*. They can be stored in three arrays, each of which is a two dimensional array with one row for each RSSI value (7 for Tinynode) and one column for each data rate (4 in our experiments). Thus the memory space required for these arrays is 28 × 3 = 84 bytes. Note that the link quality, *i.e.*, PRR, closely depends on the RSSI. Wireless transmission effects such as multi-path fading have been taken into account by the collected RSSI values. Consequently, a node only needs to maintain one copy of these three arrays. The communications of this node to its multiple neighbors can share these PRR functions (even if the radio links to different neighbors have different multi-path effect). Therefore the size of the look-up table in RA-MAC is independent of the number of neighbors and is scalable with the density of the network.

## 6. Implementation and Evaluation

In this section, we describe our experimental results using RA-MAC on the TinyNode [9] platform. We first highlight the platform’s features as well as the TinyOS [8] implementation details of RA-MAC. Then, we show the experimental results comparing the performance of RA-MAC to that of SCP-MAC. Although we chose to use TinyNode the proposed rate adaptive MAC algorithm is applicable to other off-the-shelf sensor network platforms, such as Mica2 motes (4.8 kbps to 76.8kbps), and EyesIFX nodes (4.8 kbps to 64 kbps), which support multiple transmission data rates. Recently, in fact, Atmel introduced a 900Mhz IEEE 802.15.4 transceiver (AT86RF212), which supports multiple data rates from 20 kbps to 1Mbps. Future radios will follow this trend requiring the need for algorithms such as RA-MAC.

### 6.1. TinyNode and Multi-rate Radio

Manufactured by Shockfish SA, the TinyNode platform provides a versatile ultra-low-power sensing solution for research and industrial applications. The platform includes a TinyNode 584 core device and several extension boards that support richer connectivity and storage functions. The core module TinyNode 584 is a compact device, which features an ultra low power MSP430 micro controller. The radio chip is the XE1205 [30] radio transceiver from Semtech. It can operate in the 433, 868 and 915 MHz license-free Industrial, Scientific and Medical (ISM) frequency bands.

The data rates achievable with the XE1205 radio are programmable from 1.2 kbps to 152 kbps. Lowering the transmission data rates increases the transceiver’s sensitivity extending the transmission range from approximately 150m to 1, 800m (outdoor, line of sight). It is also possible to control the range by varying the transmission power levels of sender. However, the maximum transmission power levels of transceivers have been used in most outdoor WSN applications because multi-hop transmissions may be more energy expensive compared to single-hop transmissions [2]. Therefore, we argue that varying the transmission data rates provides a more viable option for most outdoor WSN applications.

### 6.2. TinyOS Implementation

The manufacturer provides TinyOS 1.x support for TinyNodes, which includes a CSMA-style radio stack. Low Power Listening (LPL, B-MAC) [13,14] is also implemented as one of the alternatives of TinyNode MAC. B-MAC reduces receiver idle listening energy consumption by transferring the energy burden from receivers to senders, which are required to send long preambles before data packets.

By introducing scheduled channel polling, the state-of-the-art WSN MAC protocol SCP-MAC [10] can reduce B-MAC node energy consumption by ***a factor of 10***. Therefore, we decided to compare the performance of RA-MAC and SCP-MAC. Note that the rate adaptive mechanism introduced can be easily implemented with other sensor network MACs such as B-MAC and Z-MAC. However, the performance of RA-MAC will be compromised with B-MAC as the size of preamble/tone (the fixed part of Equation (9)) in B-MAC is significantly larger than in SCP-MAC.

We implement a simplified version of SCP-MAC by introducing fine-grained time synchronization on top of the existing TinyNode B-MAC component. This simplified version of SCP-MAC has almost all the features of the original except for adaptive channel polling. Because the radio duty cycles are low (e.g., one sample per minute) in long-lived WSNs, the probability of transmission collisions is small. For example, if a node transmits one packet per minute and there are 20 nodes in the radio interference range, the probability of two or more nodes transmitting simultaneously is less than 2% [29]. The simplified version of SCP-MAC, henceforth referred to as simply as SCP-MAC, is the basis of our experimental performance comparison and our proposed RA-MAC protocol.

**Figure 2.**
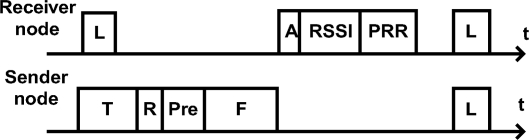
RA-MAC. **L**: Polling (11 ms). **T**: Tone(12 ms). **R**: Data rate (1 byte). **Pre**: preamble(6 bytes). **F**: data frame (34 bytes). **A**: Acknowledgement (6 bytes). **RSSI**: (1 byte). **PRR** (1 byte).

Figure 2 shows the basic operations of RA-MAC in one duty cycle. Synchronized nodes wake up and listen to the channel periodically. First, the sender transmits a tone to notify the receiver that there is one packet coming. Then, the sender transmits a one-byte data rate value, determined by Algorithm 1, to the receiver. Both tone signal and data rate value are transmitted at the base rate (*i.e.*, 9.6 kbps). Now that the receiver is ready to receive at the specified data rate (*i.e.*, either 9.6, 20, 38 or 76 kbps) the sender transmits the preamble and data frame to the receiver at the specified rate (the preamble is needed to re-calibrate signal at the new rate). After the receiver captures the whole packet, it runs Algorithm 2 to process the packet. If the packet is correctly received, the receiver transmits the ACK back to the sender using the same data rate as the data packet. Finally, the sender applies Algorithm 2 to update function *PRR*_data_ and *PRR*_ack_ upon receiving the ACK packet. The parameters values we used in the implementation are *M* = 10*, β*_1_ = 1*/*2*, β*_2_ = *β*_3_ = 1*/*32. To handle clock drift in the nodes, a synchronization packet is sent every minute at the base transmission rate. We should also note that we found that the TinyOS implementation support for 152 kbps transmission data rate was not stable, and did not use it for our RA-MAC implementation and evaluation.

**Figure 3.**
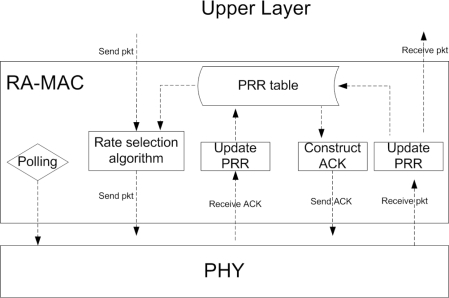
RA-MAC components in TinyOS.

Figure 3 shows the components of RA-MAC implementation in TinyOS. RA-MAC has three main components: polling, PRR table (*i.e.*, the values of function PRR(RSSI, R), which represent the relationship between PRR and RSSI & RATE) and rate selection algorithm (described in Section 5.). When the upper layer has a packet to send, RA-MAC uses the rate selection algorithm to decide the optimal data rate (which is based on PRR table). When RA-MAC receives an ACK, it extracts the PRR data that is piggybacked from the ACK packet and updates PRR table. When a transceiver (PHY component in Figure 3) receives a packet, RA-MAC calculates the PRR and updates the PRR table. If the packet is received correctly (by passing CRC check), RA-MAC constructs an ACK packet and piggybacks the PRR data in the packet before sending the packet back.

### 6.3. Goals, Metrics and Methodology

The goals of our experimental evaluation are two-fold. First, we want to study, in a single-hop environment, whether RA-MAC can reduce energy consumption compared to a fixed transmission data rate MAC protocol such as SCP-MAC. Second, we want to study the performance of the proposed RA-MAC in a multi-hop environment. We use several evaluation metrics as follows:

- *Packet delivery ratio:* packet delivery ratio is calculated as the number of packets received by the receiver divided by the number of packets sent by the sender. This metric characterizes the percentage of successful source data packet delivery; ideally, this should be 100%.
- *Network energy consumption:* this metric characterizes the network’s energy consumption at any given instant of time. We calculate the node’s energy consumption by logging the different (transmitting, listening, sleeping) states together with time stamps. The energy consumption of a radio state is calculated as a function of power consumption level (listed in Table 1) multiplied by the duration of the state. The energy consumption of a node is the summation of the energy consumption of all three states. Ideally, the energy consumption should be as low as possible.
- *Energy consumption per received packet*: this metric is calculated as a function of network energy consumption divided by the number of packets successfully received by the receiver. This metric characterizes the energy cost of successfully packet delivery. Ideally, energy consumption per received packet should be as low as possible. For ease of comparison, we normalize the energy consumption per received packet of different strategies with respect to RA-MAC. Therefore, if a normalized value is larger than 1, the corresponding strategy consumes more energy, when compared to RA-MAC, in delivering one packet from source to destination.

We ran the experiment in an outdoor environment (non-line-of-sight) for a period of 10 hours (from 10 p.m. to 8 a.m.). The period was chosen so that challenging weather conditions such as fog had a larger probability of occurring (when “midnight crisis” [3] is mostly likely to happen). The distance between a sender and a receiver is typically 50 meters. We repeated the experiments for five days using different network topology and traffic pattern scenarios, shown in the next section. For ease of comparison, we did not enable link-layer retransmissions in the experiments.

### 6.4. The Impact of Traffic Load on Network Energy Consumption

**Figure 4.**
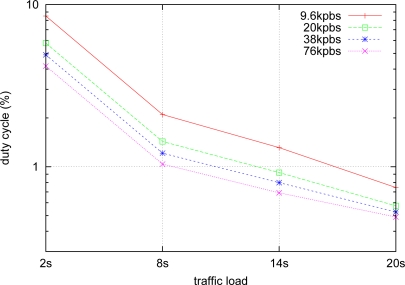
The network radio duty cycles at different traffic loads—note the log scale in Y-axis.

First, we study the applicability of the rate-adaptive algorithm at different network traffic loads. Figure 4 shows the transceiver duty cycle of nodes with different traffic loads (2, 8, 14, 20 seconds per packet respectively). Transceiver duty cycle is a popular metric to measure energy consumption in WSNs [10,11,14,18] because one can calculate transmission energy consumption by multiplying transceiver current consumption by its duty cycles. Figure 4 shows, as expected, that the network duty cycle decreases as the traffic load reduces. Further, although the network duty cycle seems to level out as the traffic load reduces (communication overheads, such as channel polling and time synchronization, become the dominating factor in network energy consumption), higher transmission data rates are more efficient in energy per packet. still consume significantly less network energy compared to lower transmission data rates. The network duty cycle is between 0.4% and 0.7% when network traffic load is low (20 seconds per packet), which is comparable to the results in [10].

### 6.5. Single Hop Experiment–High Traffic Load

**Figure 5.**
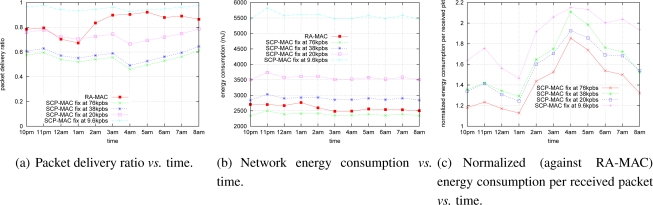
High traffic load (one packet every 2s).

**Figure 6.**
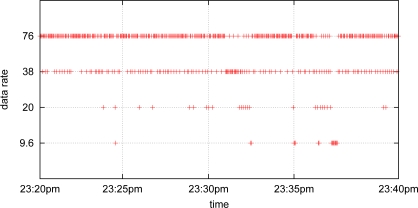
One segment of the transmission data rate value stamps.

The sender transmits one packet per two seconds in this section. Figure 5(a) shows the packet delivery ratio for different strategies (fixed rates and RA-MAC). As expected, lower transmission data rates performed better than higher transmission data rates. RA-MAC operated between 9.6 and 20 kbps. Figure 5(b) illustrates the energy consumption of different strategies. It shows that higher transmission data rates consumed less energy than the lower transmission data rates. It is interesting to note that RA-MAC performed between 38 kbps and 76 kbps in this metric, which is different to Figure 5(a). In summary, RA-MAC performed closed to the better fixed rate strategies in both cases. Figure 5(c) shows that RA-MAC significantly outperformed all fixed rate strategies (by more than 200%) compared with 9.6 and 38 kbps at 4am. Figure 5(c) also shows that RA-MAC consumes minimum energy to successfully deliver a packet. Figure 6 plots the transmission data rate stamps in one segment of the experiment (20 minutes). It shows that RA-MAC is adapting to the channel condition dynamically with different transmission data rates.

### 6.6. Single Hop Experiment (Low Traffic Load)

**Figure 7.**
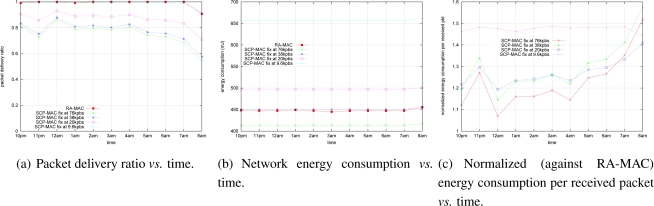
Low traffic load (one packet every 20s).

We repeated the experiment in Section 6.5. with significantly lower traffic load (one packet every 20 seconds); Figure 7 shows the results. Overall, the results under low network traffic load are similar to those under high network traffic load (see Section 6.5.). RA-MAC performed close to the best fixed rate strategies in term of both packet delivery ratio (Figure 7(a)) and network energy consumption (Figure 7(b)). RA-MAC significantly outperformed all fixed rate strategies in the energy consumption per successful packet delivery (Figure 7(c)).

It is worth noting that the link quality in Section 6.5. is different to that in Section 6.6. RA-MAC managed to outperform fixed rate SCP-MAC under different link quality and traffic loads, which shows the robustness and applicability of RA-MAC.

### 6.7. Multi-hop Experiment

**Figure 8.**
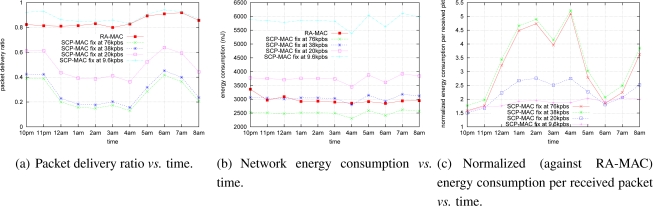
Two hops network (one packet every 4s).

In this section, we evaluate the performance of RA-MAC in a 2-hop network. We used static routing, where a sender sends a packet to a receiver via a forwarder every four seconds in this experiment. The results are shown in Figure 8. Compared to the one-hop network in Sections 6.5. and 6.6., RA-MAC reduced network energy consumption compared to a fixed-rate SCP-MAC even further in a two-hop environment (maximum reduction of 5 times at 4am in Figure 8(c) instead of 2.2 times at 4pm in Figure 8(c)). This behavior can be explained by significantly lower end-to-end packet delivery rates at high transmission rates of the two-hop network (Figure 8(a)) than the packet deliver rates of the one-hop network observed during the experiments Figure 5(a)).

## 7. Comparison with WLAN Rate-adaptive MAC by Simulations

In this section, we compare RA-MAC with a popular technique used for WLAN (see Section 2.2.). We presents a simulation comparison between conventional WLAN rate adaptive algorithm—Auto Rate Fallback (ARF) — and proposed RA-MAC rate adaptive algorithm introduced in Section 5. In the WLAN rate adaptive algorithm [22], the sender increases transmission data rates after ten consecutively successful packet delivery, and decreases transmission data rates after one failed packet delivery.

We used an experimental log file, which records packet data rates, packet delivery status (success or failure), RSSI values, node radio states, and time-stamps, collected by the TinyNodes to calculate the packet delivery ratio, energy consumption and energy consumption per received packet (same metric as Section 6.3.). Based on packet delivery status and RSSI values recorded in the log file, ARF and RA-MAC uses different strategies to choose transmission data rates. The probability of successful delivery given a certain RSSI value, and the average amount of energy consumed given a specific transmission data rate, are based on the statistics over the whole experiment.

**Figure 9.**
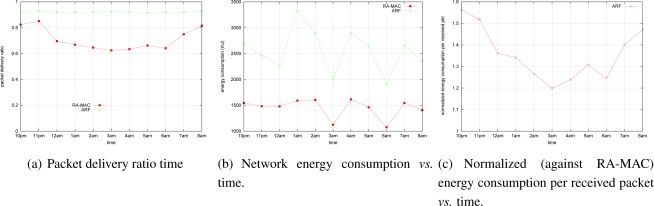
RA-MAC *vs.* ARF.

Although ARF achieved higher link packet delivery ratio compared to RA-MAC (Figure 9(a)), it consumed higher network energy (Figure 9(b)). Overall, Figure 9(c) shows that RA-MAC, compared to ARF, achieved significantly higher transmission energy consumption efficiency by consuming less energy for one successful delivery. The rate-adaptive algorithms in WLAN (e.g., ARF) has focused on optimizing network throughput which is important to users of WLANS. However this may not be applicable to the context of WSN, in which energy is usually a more important metric.

The link quality in WSNs is typically more dynamic and lossy than that in WLAN. WSN nodes are usually equipped with inexpensive radio transceivers, with poorer gain and SNR. Many WSN nodes use narrow band FSK communications which is more susceptible to fading than spread spectrum modulation techniques. WSN nodes have a significantly smaller transmission energy budget compared to WLAN nodes because of network lifetime concerns. Finally, WSNs are often deployed in harsh, or even hostile, environments with significant attenuation and multi-path fading. Consequently, it is difficult to achieve 10 consecutive successful transmissions, and ARF had to use low transmission data rates most of the time.

## 8. Conclusion

We have introduced RA-MAC, an energy-efficient Rate Adaptive Media Access Control protocol for long-lived WSNs. Previous research has shown that the dynamic, lossy, and asymmetric nature of wireless communications is one of the major challenges to reliable data delivery in WSNs. This is due to nodes operating within the transition zone where the received SNR is highly variable and various noise processes contribute to the unreliable behavior. RA-MAC increases the link quality by raising the SNR, trading it off against data bit rate which generally far exceeds the requirement of the link. We have used an analytical model of energy consumption at different data rates and channel conditions to ***minimize network energy consumption***. Finally, we have implemented RA-MAC in TinyOS for an off-the-shelf sensor platform (the TinyNode), and compared its performance with the state-of-the-art WSN MAC Protocol (SCP-MAC) in both simulation and experiment, showing the real benefits of our protocol.

## Figures and Tables

**Table 1.** Mathematical notations and their values used in theoretical analysis and experiment.

**Symbol**	**Definition**	**Theory**	**Experiment**
PRR	Packet Reception Rate	Varying	Varying
BER	Bit Error Rate	Varying	Varying
SNR	Signal to Noise Ratio	Varying	Varying
RSSI	Receiver Signal Strength Indicator	Varying	Varying
*f_data_*	Date frame size in bits	272	272
*f_ack_*	ACK frame size in bits	64	64
*E_b_*	The average energy per bit	Varying	Varying
*N*_0_	Noise power spectral density	Varying	Varying
*R*	Data bit rate	1.2 to 152 kbps	9.6, 20, 38, 76 kbps
*B*	Channel bandwidth	Varying	Varying
*N*	Channel noise power	Varying	Varying
*k*	Boltzmann’s constant	1.38 × 10^−23^ J/K	1.38 × 10^−23^ J/K
*T*	Effective temperature in Kelvin	Varying	Varying
*E*	Node energy consumption	Varying	Varying
*n*	The number of neighbors	10	1
*P_l_*	Power in listening	2.85 mA	2.85 mA
*P_tx_*	Power in transmitting	25.4 mA	25.4 mA
*P_rx_*	Power in receiving	15.1 mA	15.1 mA
*t_l_*	Listening time	11 ms	11 ms
*t_tone_*	Polling time	12 ms	12 ms
*α*	One hop link reliability requirement	1	1

## References

[1] Langendoen K., Baggio A., Visser O. (2006). Murphy Loves Potatoes: Experiences from a Pilot Sensor Network Deployment in Precision Agriculture.

[2] Selavo L., Wood A., Cao Q., Sookoor T., Liu H., Srinivasan A., Wu Y., Kang W., Stankovic J., Young D., Porter J. (2007). LUSTER:Wireless Sensor Network for Environmental Research.

[3] Dinh T.L., Hu W., Sikka P., Corke P., Overs L., Brosnan S. (2007). Design and Deployment of a Remote Robust Sensor Network: Experiences from an Outdoor Water Quality Monitoring Network.

[4] Chen Q., Hu W., Corke P. (2009). Poster Abstract: Energy-efficient Rate Adaptive MAC Protocol (RA-MAC) for Long-lived Sensor Networks.

[5] Anastasi G., Borgia E., Conti M., Gregori E., Passarella A. (2005). Understanding the Real Behavior of 802.11 and Mote Ad hoc Networks. Pervasive Mob. Comput.

[6] Zamalloa M., Krishnamachari B. (2007). An Analysis of Unreliability and Asymmetry in Low-power Wireless Links. ACM Trans. Sens. Netw.

[7] Zhao J., Govindan R. (2003). Understanding Packet Delivery Performance in Dense Wireless Sensor Networks.

[8] TinyOS http://www.tinyos.net/.

[9] Dubois-Ferrière H., Fabre L., Meier R., Metrailler P. (2006). TinyNode: A Comprehensive Platform for Wireless Sensor Network Applications.

[10] Ye W., Silva F., Heidemann J. (2006). Ultra-low Duty Cycle MAC with Scheduled Channel Polling.

[11] Ye W., Heidemann J., Estrin D. (2002). An Energy-efficient MAC Protocol for Wireless Sensor Networks.

[12] van Dam T., Langendoen K. (2003). An Adaptive Energy-efficient MAC Protocol for Wireless Sensor Networks.

[13] Hill J., Culler D. (2002). Mica: A Wireless Platform for Deeply Embedded Networks. IEEE Micro.

[14] Polastre J., Hill J., Culler D. (2004). Versatile Low Power Media Access for Wireless Sensor Networks.

[15] Buettner M., Yee G.V., Anderson E., Han R. (2006). X-MAC: A Short Preamble MAC Protocol for Duty-cycled Wireless Sensor Networks.

[16] Barroso A., Roedig U., Sreenan C.J. (2006). f-MAC: A Deterministic Media Access Control Protocol Without Time Synchronization.

[17] Halkes G.P., Langendoen K. (2007). Crankshaft: An Energy-Efficient MAC-Protocol for Dense Wireless Sensor Networks.

[18] Rhee I., Warrier A., Aia M., Min J. (2005). Z-MAC: A Hybrid MAC for Wireless Sensor Networks.

[19] Burri N., von Rickenbach P., Wattenhofer R. (2007). Dozer: Ultra-low Power Data Gathering in Sensor Networks.

[20] Sun Y., Du S., Gurewitz O., Johnson D. (2008). DW-MAC: A Low Latency, Energy Efficient Demand-wakeup MAC Protocol for Wireless Sensor Networks.

[21] Liu S., Fan K.W., Sinha P. (2009). CMAC: An Energy-efficient MAC Layer Protocol using Convergent Packet forwarding for Wireless Sensor Networks. Trans. Sens. Netw. (TOSN).

[22] Kamerman A., Monteban L. (1997). WaveLAN-II: a High-performance Wireless LAN for the Unlicensed Band. Bell Labs Tech. Jour.

[23] Lacage M., Manshaei M.H., Turletti T. (2004). IEEE 802.11 Rate Adaptation: A Practical Approach.

[24] Holland G., Vaidya N., Bahl P. (2001). A Rate-adaptive MAC Protocol for Multi-Hop Wireless Networks.

[25] Sadeghi B., Kanodia V., Sabharwal A., Knightly E. (2002). Opportunistic Media Access for Multirate ad hoc Networks.

[26] Haratcherev I., Taal J., Langendoen K., Lagendijk R., Sips H. (2005). Automatic IEEE 802.11 Rate Control for Streaming Applications: Research Articles. Wirel. Commun. Mob. Comput.

[27] Qiao D., Choi S., Jain A., Shin K.G. (2003). MiSer: An Optimal Low-energy Transmission Strategy for IEEE 802.11a/h.

[28] Woo A., Tong T., Culler D. (2003). Taming the Underlying Challenges of Reliable Multihop Routing in Sensor Networks.

[29] Le T., Hu W., Corke P., Jha S. (2009). ERTP: Energy-efficient and Reliable Transport Protocol for Data Streaming in Wireless Sensor Networks. Comput. Commun.

[30] XE1205 Radio Transceiver Datasheet www.semtech.com/pc/downloadDocument.do?navId=H0,C1,P2615&id=769/.

